# Inclusive Rehabilitation Process Ensuring the Best Outcome for Post Injury Intervention – A Social Security Perspective

**DOI:** 10.21315/mjms-01-2026-s01

**Published:** 2026-04-30

**Authors:** Mohammed Azman Aziz Mohamed, Azlan Darus, Hafez Hussain

**Affiliations:** 1 Social Security Organisation (PERKESO), Kuala Lumpur, Malaysia; 2 International Social Security Association (ISSA), Geneva, Switzerland; 3 CEO Tersohor, School of Medical Sciences, Universiti Sains Malaysia, Health Campus, Kelantan, Malaysia; 4 Elite Fellow, Faculty of Medicine, University Malaya, Kuala Lumpur, Malaysia

**Keywords:** rehabilitation, social security, return to work, PERKESO, disability management, occupational injury

## Abstract

Injuries remain a significant contributor to disability and economic burden globally and in Malaysia. Traditional models of care often position rehabilitation as a late-stage intervention, limiting functional recovery and return-to-work outcomes. This paper presents a social security-driven approach to rehabilitation, drawing on the experience of the Social Security Organisation of Malaysia (PERKESO). Emphasis is placed on early intervention, integrated case management, investment in advanced rehabilitation technologies, and sustainable financing mechanisms. Evidence from PERKESO’s rehabilitation approaches, infrastructure and disability management processes demonstrate improved reintegration outcomes and functional recovery. This model highlights the importance of embedding rehabilitation across the continuum of care to optimise outcomes and strengthen workforce productivity.

## Introduction

Injuries pose as one of the main reasons for hospitalisation and medical care in the country. Despite various efforts and resources allocated to other main reasons of hospitalisation, especially non-communicable diseases, injuries, and accidents remain a mainstay of causes of disability in the country and unfortunately affect a significant portion of the country’s resources and economic impact (1, 2). As the social security organisation covering the workers in the country, Social Security Organisation of Malaysia (PERKESO) recorded firsthand the impact of injuries on the working population of the country (3).

In the field of social security, the impact of these accidents exceeded the acute period of injury when treatment is required. The sequelae of the injuries persisted, in most cases permanently, in the form of permanent disablement (4).

The goal of treatment is always to be able to return the patient as closely as they can to the functional ability prior to the incident. Therefore, surviving the incident and discharged from care alone is insufficient for long term social security (1).

PERKESO recorded a high number of invalidity applications annually, exceeding 42,000 applications in the year 2025. This translates as workers who reported inability to work permanently in a year, a significant loss to the labour force of the country. On the other hand, 71,404 workers claimed injuries for employment in the same year, a significant portion of whom were compensated for permanent disablement. Regardless that some degree of impairment following injuries may be expected, it is pertinent that the resulting impairment must be minimised as much as possible. Throughout the treatment journey, the rehabilitation process should be regarded as an important component (3).

## Rehabilitation in the Chronology of Post Injury Care

The model for rehabilitation in the chronology of care has always been following a set protocol, where the rehabilitation process is usually concentrated post discharge. This model, introduced way back in 1946, has been sufficient for some time; however, it is riddled with a few challenges (4). The experience of PERKESO monitoring our insured persons throughout their journey of care revealed several gaps that needed to be addressed.

### Rehabilitation Introduced Later in Care

The model of care usually introduces rehabilitation (intensive or outpatient basis) later in the treatment phase. Although allied health procedures such as physiotherapy may be prescribed during inpatient care, this is usually in support of the clinical care rather than preparing the patient for the post discharge period, and ultimately the return-to-work period later. Rehabilitation is usually regarded in the tertiary prevention period and hence may only be introduced to the patient later (1).

### Loss to Follow Up

Rehabilitation process is also exposed to loss to follow up of the patient, due to various reasons such as accessibility, mobility and transportation, motivation and return-to-work support and lack of clear goals. Loss to follow up not only delays recovery but also hinders optimal recovery due to opportunity loss for recovery at the appropriate time post injury (5). Complications such as muscle atrophy, spasticity, joint stiffness or reduced range of movement will hinder rehabilitation goals and will be a further psychological hindrance for the best possible outcome.[Fig f1-01mjms3302_ed]

### Insufficient Rehabilitation: Technology and Modality

The advent of technology in rehabilitation is far advanced compared to a decade ago. Use of technology such as neural rehabilitation, robotic, gamification and biopsychosocial approaches to rehabilitation is shown to be able to alter positively the outcome of treatment following injury (2).

The gap in the country lies in the availability of these rehabilitation modalities to the general public. These gaps lie in two main factors:

- Availability of the technology is currently only in major hospitals or specific rehabilitation private hospitals. Therefore, patients outside the coverage of the centres are at risk of missing out on the comprehensive rehabilitation and falling into the insufficient rehabilitation gap.
- Financial coverage – rehabilitation more often than not is not covered within the private medical insurance in the country (1). Although inpatient rehabilitation is covered within the hospitalisation cost, intensive rehabilitation post discharge is still borne out of pocket or by private financiers such as the employer. In this role, PERKESO frequently serves as the financier for the rehabilitation process for the working population insured by the organisation.

## The Approach to Rehabilitation: Social Security

Ensuring social security of the working population does not only mean that the injured workers are fairly and efficiently compensated. Optimal outcomes are achieved not merely through compensation but through restoring functional independence and enabling reintegration into the workforce (6). The organisation views that the best form of social security is for the injured workers to be able to return to their functional capacity as a productive member of the society (7).

### The Concept of Rehabilitation as an Early Intervention

Rehabilitation should be planned and started at the earliest part of the treatment journey. Therefore, involvement of the rehabilitation physician and case management requires comprehensive planning and resources. The concept of rehabilitation as a tertiary prevention needed to be revised and introduced into secondary prevention at the inpatient hospital care (4). The organisation is cognisant of this factor and has introduced an early intervention programme involving our case managers nationwide. Patients who will qualify for social security support for rehabilitation and case management are identified early in their claim process. The triaging was conducted efficiently via artificial intelligence, which increases the capacity for case management significantly. The concept also reduces the gap in rehabilitation mentioned earlier, where early recognition reduces loss to follow up and starts earlier. Figure 2 illustrates our suggested approach to the levels of prevention where rehabilitation is introduced early. The proof of concept for such approach is proven with the care post myocardial infarction, where cardiac rehab is introduced early and comprehensively as part of the recovery process (1).

### Building Resources

Since 2014, the organisation has been operating the largest Rehabilitation Centre in South East Asia, located in Malacca (Figure 3). The 381-bed rehabilitation centre provided comprehensive rehabilitation and vocational training to enable the optimal recovery for injured patients. In order to provide wider coverage, the organisation had also established multiple satellite centres and strategic partners throughout the country that will be able to provide equivalent rehabilitation services (7). To enable comprehensive coverage nationwide, the organisation have also recently completed the establishment of a larger world-class rehabilitation centre in Ipoh (Figure 4), and is currently constructing the third centre in Terengganu (Figure 5). Further plan for centres in East Malaysia is also already in the pipeline.

### Investment in Advanced Rehabilitation Technology

To achieve the best possible outcome, PERKESO, through its Rehabilitation Centres, had also invested in advanced technological modalities of rehabilitation. Central to this is the Cyberdyne Technology from Japan, utilising the Hybrid Assistive Limb exoskeleton for upper and lower limb rehabilitation (8).

Other new technologies were also introduced, including robotic rehabilitation and advanced therapy modalities such as ROBERT^®^, Lightforce Laser Therapy and MetaMotus Galileo, just to name a few. These technologies serve as adjuvants to other world-class facilities such as advanced hydrotherapy, simulated vocational therapy, carer training and carer facility, advanced recreational facility and multitude of other technologically advanced facilities available, that the rehabilitation centre is aptly known as the National Neuro-Robotics and Cybernics Centre. The investment in high-end technology signifies the organisation’s intention that rehabilitation is an integral part of the patient journey, positioning the centre as a national neuro-rehabilitation hub (9).

### Financing Rehabilitation

Financing for rehabilitation remains a large hurdle for patients, particularly since intensive rehabilitation post discharge is not readily covered via private or employment insurance. A large proportion of the worker population, therefore, relies on the services provided by government hospitals, while those who are able to manage through out-of-pocket payment to private centres.

PERKESO, however, are mandated through the Employees Social Security Act to provide rehabilitation services to its insured persons. The services provided at the PERKESO Rehabilitation Centres are free for the injured persons (3).

A study by ISSA on return on investment on reintegration yielded positive return for every dollar spent in rehabilitation and return-to-work programmes (10).[Fig f6-01mjms3302_ed]

## Case Management and Employment Services

Central to rehabilitation of patients, PERKESO emphasises the process of comprehensive case management. Clinical rehabilitation is complemented by a multi-disciplinary involvement from social security, the clinical team, the patient and their carers and also the employer (11). The organisation applies a triage system using artificial intelligence, and coupled with the organisation’s role as a national public employment services provider, the programme enables a higher rate of return or retain at work for workers. This approach enables improved return-to-work outcomes for workers who are injured (12).

## Conclusion

Returning injured workers to the best of their pre-injury functions is the most essential goal for treatment and rehabilitation. For decades, clinical pathways have focused on acute medical intervention, while comprehensive rehabilitation had always taken place later as tertiary prevention measures (4). Therefore, despite surviving the injury or disease, the patients lacked the ability to be able to fully function as a productive member of the society; moreover, those who sustain inability to work or earn their living. The Social Security Organisation of Malaysia recognise an important element in the clinical pathway of treatment of the worker should involve comprehensive rehabilitation measures. This, however, will only be of value if:

- The rehabilitation process is introduced early into the treatment regime.
- The rehabilitation is sustained over the length of treatment, with the risk for treatment attrition to be managed through case management.
- The rehabilitation includes comprehensive modalities and state-of-the-art technology to regain most functions that are lost through injury or diseases.
- The access to such rehabilitation services is made possible through the presence of modern facilities and widespread locations.
- The financial model for rehabilitation is ascertained so as to be accessible to all patients.

## Figures and Tables

**Figure 1 f1-01mjms3302_ed:**
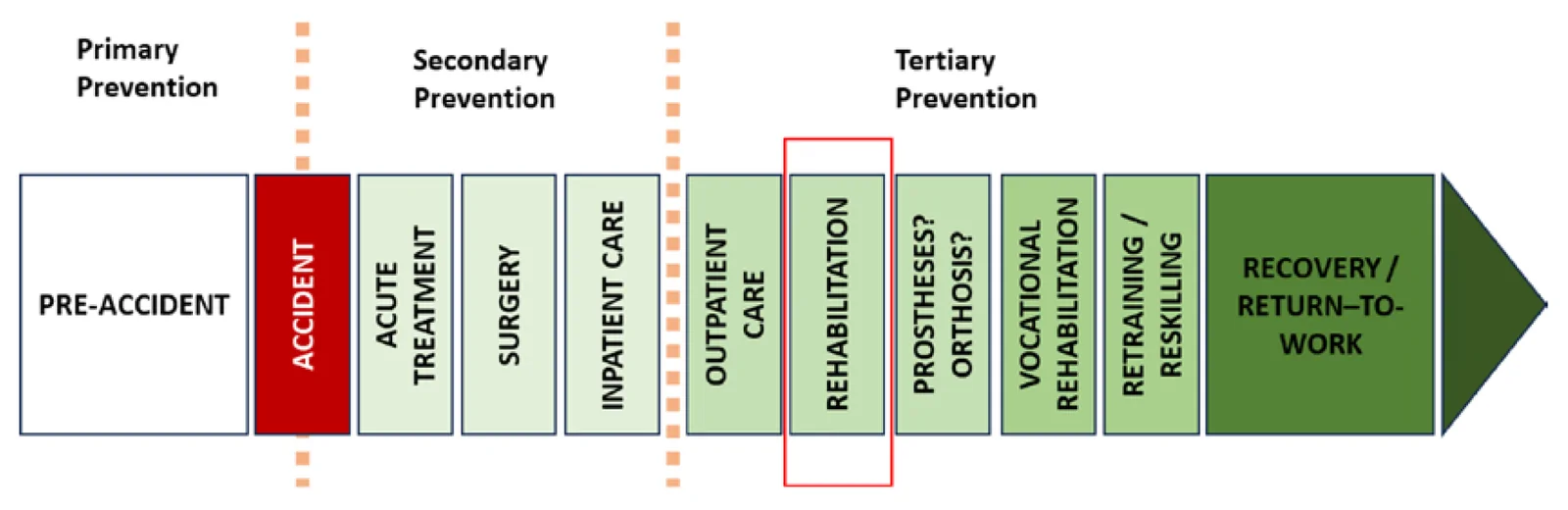
Levels of prevention (adapted from the Non-Communicable Diseases model, 1946)

**Figure 2:**
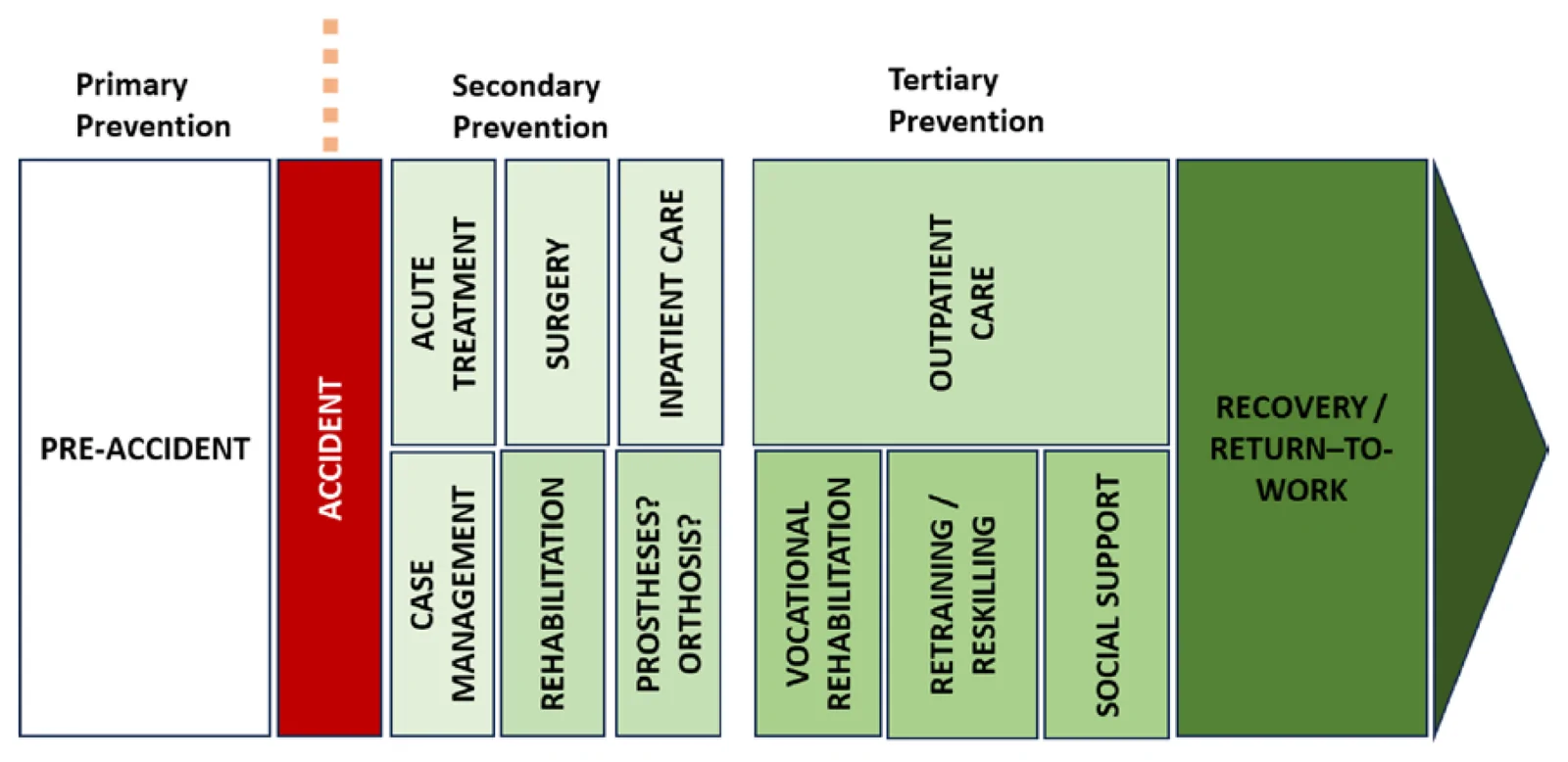
Integrated prevention levels

**Figure 3:**
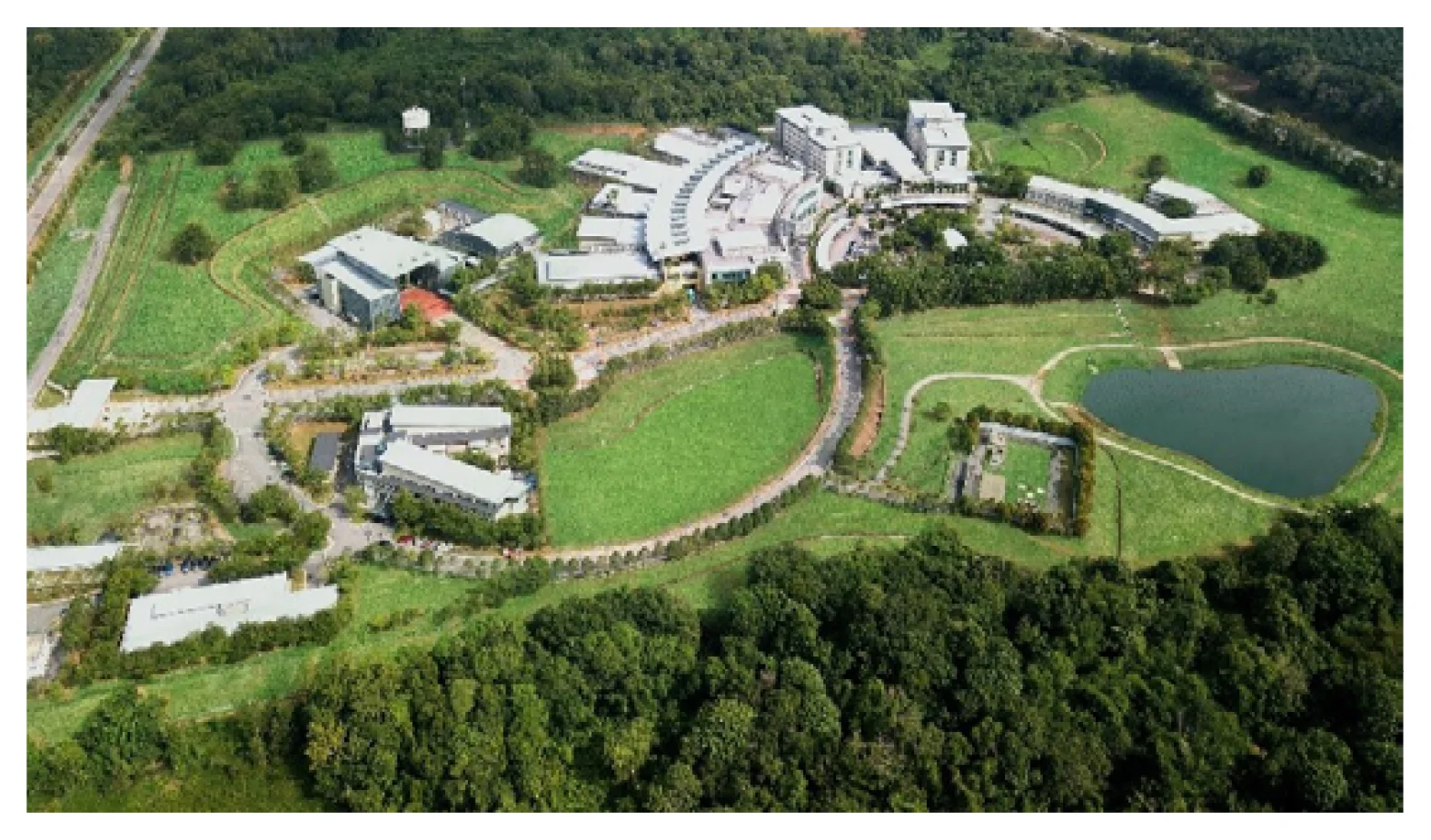
Pusat Rehabilitasi PERKESO Tun Abdul Razak, Malacca (launched 2014)

**Figure 4:**
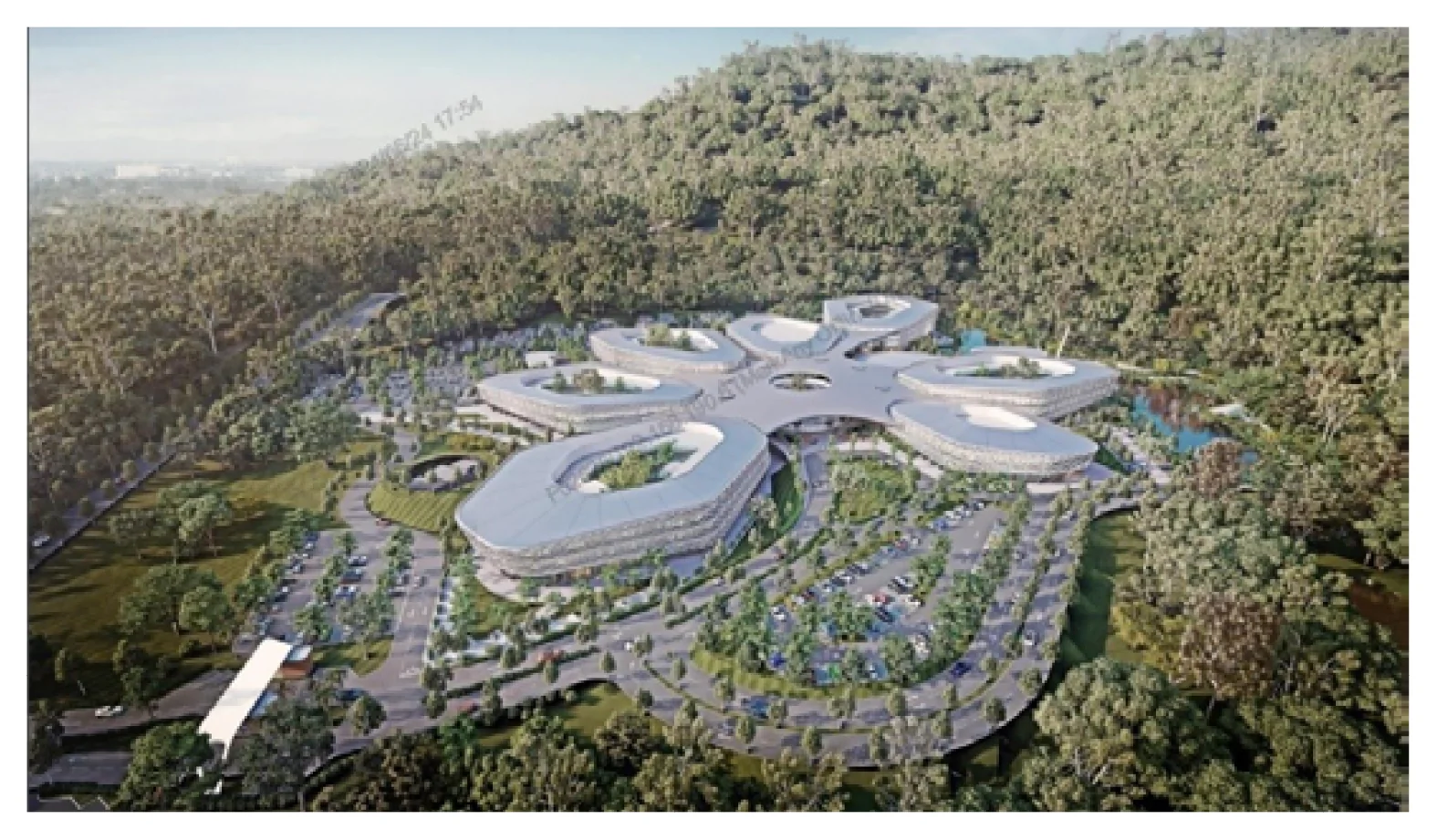
Pusat Rehabilitasi PERKESO Ipoh, Perak (to be launched 2026)

**Figure 5:**
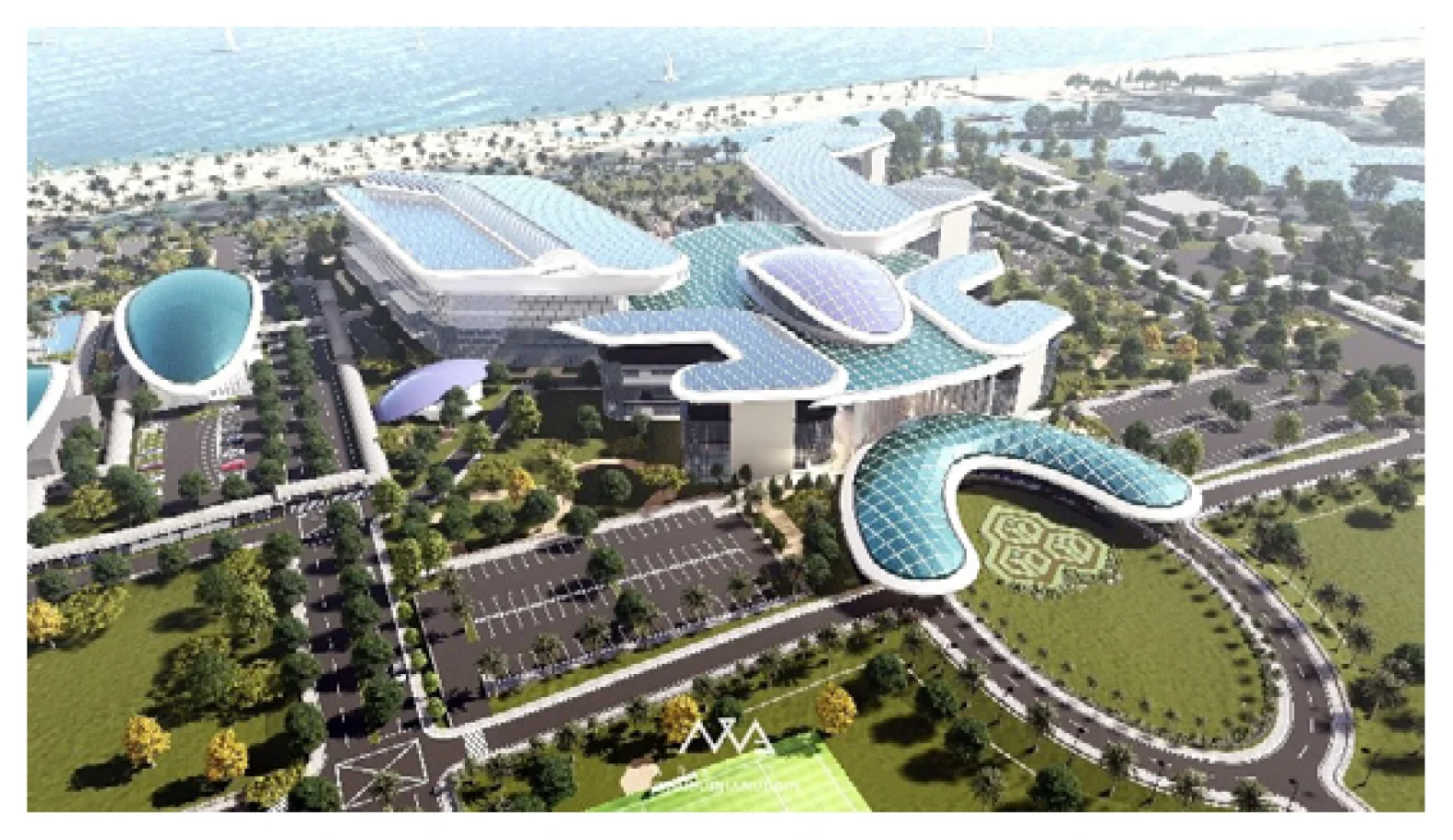
Pusat Rehabilitasi PERKESO Terengganu (under construction, 2027)

**Figure 6 f6-01mjms3302_ed:**
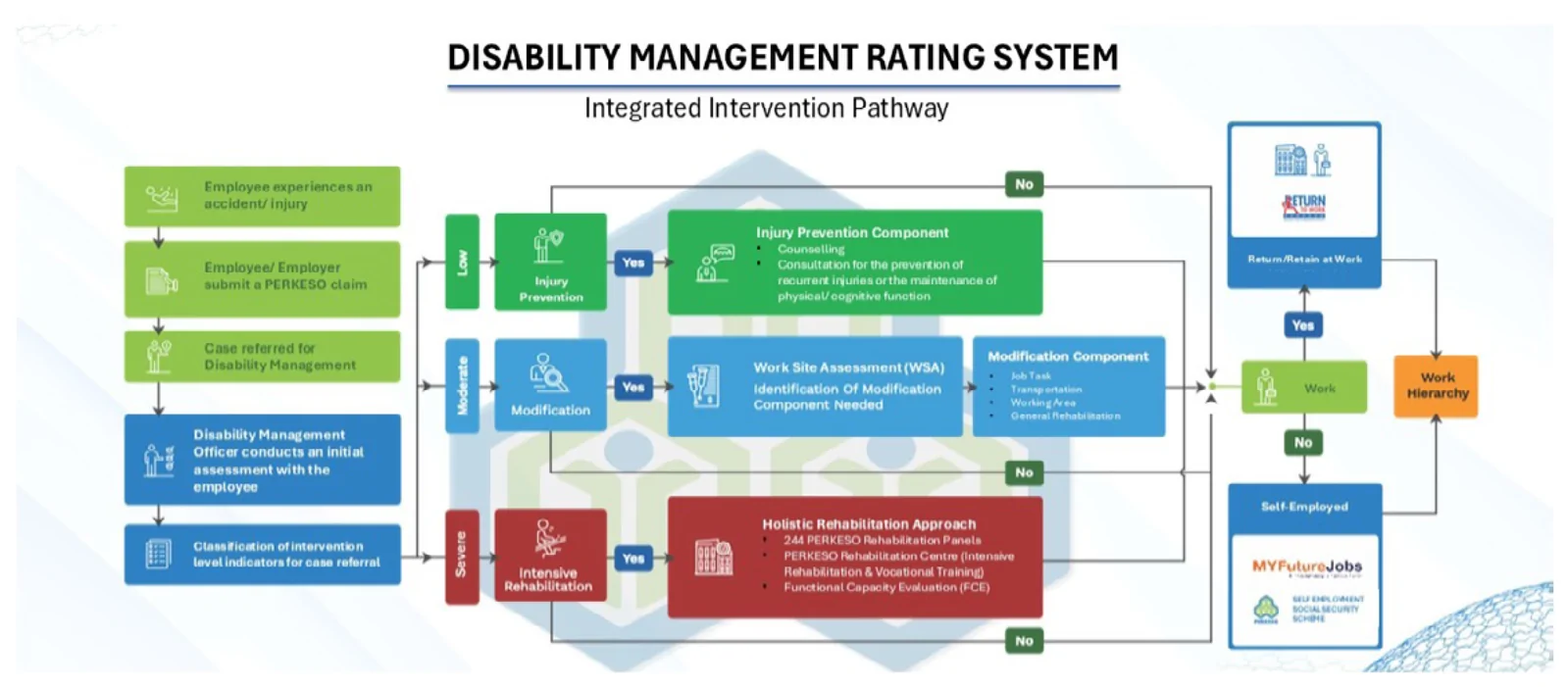
A comprehensive case management model for rehabilitation by PERKESO
